# In Situ Prediction of Microstructure and Mechanical Properties in Laser-Remelted Al-Si Alloys: Towards Enhanced Additive Manufacturing

**DOI:** 10.3390/ma17143622

**Published:** 2024-07-22

**Authors:** Metin Kayitmazbatir, Mihaela Banu

**Affiliations:** Mechanical Engineering Department, University of Michigan, 2350 Hayward, Ann Arbor, MI 48109, USA; metink@umich.edu

**Keywords:** laser remelted Al-Si alloys, strengthening mechanism, in-situ prediction

## Abstract

Laser surface remelting of aluminum alloys has emerged as a promising technique to enhance mechanical properties through refined microstructures. This process involves rapid cooling rates ranging from 10^3^ to 10^8^ °C/s, which increase solid solubility within aluminum alloys, shifting their eutectic composition to a larger value of silicon content. Consequently, the resulting microstructure combines a strengthened aluminum matrix with silicon fibers. This study focuses on the laser scanning of Al-Si aluminum alloy to reduce the size of aluminum matrix spacings and transform fibrous silicon particles from micrometer to nanometer dimensions. Analysis revealed that the eutectic structure contained 17.55% silicon by weight, surpassing the equilibrium eutectic composition of 12.6% silicon. Microstructure dimensions within the molten zones, termed ‘melt pools’, were extensively examined using Scanning Electron Microscopy (SEM) at intervals of approximately 20 μm from the surface. A notable increase in hardness, exceeding 50% compared to the base plate, was observed in the melt pool regions. Thus, it is exemplified that laser surface remelting introduces a novel strengthening mechanism in the alloy. Moreover, this study develops an in situ method for predicting melt pool properties and dimensions. A predictive model is proposed, correlating energy density and spectral signals emitted during laser remelting with mechanical properties and melt pool dimensions. This method significantly reduces characterization time from days to seconds, offering a streamlined approach for future studies in additive manufacturing.

## 1. Introduction

Aluminum (Al) alloys have been the subject of extensive research for various applications, especially within the automotive and aerospace industries [1,2,3]. Aluminum alloys containing silicon (Si) in varying amounts have also attracted ongoing interest, particularly those that fall within the hypo-eutectic range (below 12.6% in weight). However, attention has shifted over time towards eutectic and hyper-eutectic alloys, owing to their superior strength–weight ratio, exceptional machinability, and impressive wear resistance [4,5]. The superior strength–weight ratio is attributed to the fact that in these alloys, Si fibers are embedded in an Al matrix, and the resulting material exhibits similarities to a composite material with the difference that the reinforcement (Si density 2.35 g/cm^3^ Si) are lighter than the matrix (Al density 2.70 g/cm^3^) [6]. A higher volume fraction of Si improves the strength–weight ratio, thus researching how to exceed the equilibrium eutectic structure 12.6% weight of Si is of high interest. One way to increase the percentage of Si is to shift the eutectic point towards a higher Si composition [7]. It was demonstrated that extremely high cooling rates (10^3^–10^8^ °C/s) obtained in laser processing have this effect on Al-Si alloys and moreover, the resulting mechanical properties, including fracture toughness, hardness, and tensile strength are improved [7,8,9]. This correlation between the cooling rates and improving the mechanical properties is available only for up to a certain threshold from which Si nucleation begins to form agglomerated polygonal shapes, degrading the mechanical properties and adversely affecting the overall mechanical properties. So, a technique is needed to enhance the Si content and refine the microstructures of Al-Si alloys while avoiding the agglomeration of Si.

Laser surface remelting is an effective method for achieving this effect, as it allows precise control over alloys’ solidification parameters. By adjusting these parameters, one can achieve specific desired results, leading to enhanced mechanical properties and better performance of the alloy. In this paper, we proposed a method to create correlations between the laser melting parameters and the mechanical properties of the resulting materials by using an in situ plasma analyzer. The collection of in situ plasma signals from laser processes using spectral analysis captures the plasma signal produced by the interaction between the laser and the material, which contains various indicators. These indicators are crucial for predicting the final state of the processed material. Research has explored applications such as composition analysis [10], detecting changes in material hardness [11,12], observing phase transformations during processing [13], and identifying voids in the heat-affected zone [14,15,16]. A particularly valuable feature of this technique is its immediacy, providing rapid and reasonably accurate predictions about material properties, which is essential for reducing the time required for material characterization. Conventional characterization often involves destructive testing, which can make laser-processed materials more costly. Thus, one objective of the current study is to establish a correlation between the in situ plasma signals and the dimensions of the melt pool. The results could enable immediate feedback, potentially contributing to closed-loop control in the future.

In this study, Al-20Si by weight specimens, i.e., Al–20Si (wt%) were produced through arc melting and then subjected to laser remelting. As mentioned in the previous paragraph, the research aimed to investigate the correlation between processing parameters and the resulting microstructures of the Al-Si alloy created under high cooling rates. The effects of both super-saturated and refined eutectic structures were assessed within hardness testing. Moreover, the plasma signals from the laser–material interaction were continuously monitored and analyzed for spectral signals. Specifically, optical emission spectral data in the range of 320 nm to 410 nm were collected, as these are typical wavelengths at which metal plasma emissions are observed. These collected signals were then analyzed using the Smart Optical Manufacturing Systems (SOMS) software to characterize the microstructure and approximate the melt pool dimensions. By capturing and analyzing the plasma signals during laser processing, the method aims to predict microstructural changes, offering immediate feedback for enhanced alloy performance and efficiency in material characterization compared to the traditional destructive testing methods.

## 2. Materials and Experimental Setup

**Figure 1:**
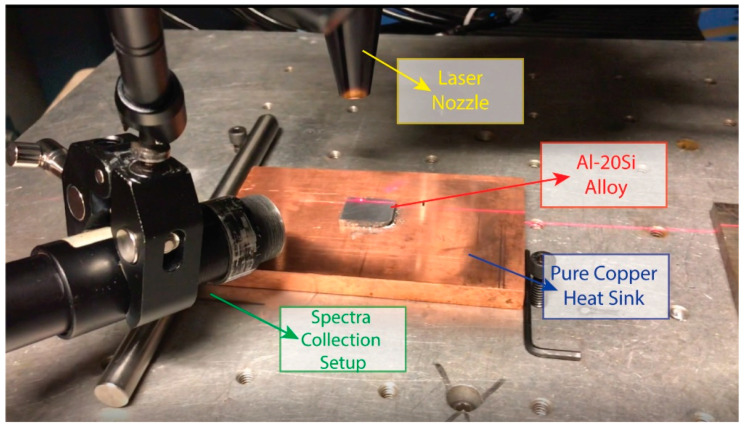
Experimental setup for laser processing and spectral signal collection.

A bulk arc-melted Al-20Si (wt%) alloy (in weight percent) produced at Ames National Lab was subsequently cut into smaller samples measuring 22.25 mm in length, 17 mm in width, and 4.5 mm in thickness. These samples were then ground using 1200-grit SiC sandpaper, resulting in a mirror-like surface on both sides. These samples were attached to a copper block using a thermally conductive paste with a conductivity of 73 W/mK. A disk laser (Trumpf HLD 4002, Trumpf Laser, Plymouth, MI, USA), which operates at a wavelength of 1030 nm, was utilized to remelt the surface of the material with a 60 standard cubic feet per hour Argon flow. The laser beam diameter was consistently fixed at 0.6 mm for all the experiments. The experimental setup is presented in Figure 1.

The plasma produced during the interaction between a laser and the material was monitored through lenses which were attached to the laser head at a 2 mm distance above the laser spot. The signals obtained were transferred to the SOMS spectroscope with an optical fiber cable. This spectroscope houses 2048 CCD units and operates with an integration time of 10 ms. For additional processing, the spectroscope was connected to a computer.

The experimental parameters which were varied during the experiments are scanning speed, 30–180 mm/s; laser power, 1200–1500 W; and a fixed 0.6 mm laser beam diameter. The Al-20Si (wt%) samples of 4.5 mm and having an initial roughness of 9 µm were placed on a heat sink made of copper. The spectroscopy system was set up with the spectral head positioned 2 mm above the gantry-mounted laser head. The spectrometer was configured with an integration time of 10 ms and a resolution of 0.02 nm.

The design of the experiments included two critical process parameters: laser power (W) set at 1200 and 1500 (2 levels), and laser scanning speed (mm/s) set at 30, 60, 90, 120, 160, and 180 (6 levels), resulting in a total of 12 energy inputs for melting. Laser power variations primarily influence the melting energy, while laser scanning speed affects solidification rates. The experimental design was constrained by the availability of materials; it utilized an arc-melted and homogenized material suitable for laser melting, limiting our sample sizes. Consequently, it adopted a fractional factorial analysis, utilizing a complete dataset only for laser power at 1200 W across all 6 levels of scanning speed and three other critical values obtained through a combination of laser power at 1500 W and laser scanning speed at 60 mm/s, conditions near the material’s vaporization limits. 

Experiment #7, which employed a higher laser power (1500 W) than the other initial tests, was designed to probe conditions at the brink of vaporization. Table 1 summarizes the experimental conditions.

These specific parameters were chosen because they significantly influenced the deposited energy and consequently affected the mechanical properties of the final material [7]. The control group experiments were conducted on off-the-shelf Al5052 plates with 50 different parameters.

The melt pools obtained within this series of seven combinations were analyzed through cutting 5 mm × 5 mm samples, mounting them in an epoxy resin, followed by grinding, polishing, and etching. Scanning Electron Microscope (SEM) Tescan MIRA3, Michigan USA the FEG in secondary electron mode for microstructure morphology analysis and Electron Backscatter Diffraction (EBSD) for grain sizes were used for these samples. A MATLAB script was used for the identification of the eutectic structures of the samples, focusing on their minimum, maximum, median, and average sizes. Further, the hardness of the base material and the remelted zone produced by the laser was measured using a Clark digital micro hardness tester model CM-400AT, Michigan, USA. Since the overall hardness did not noticeably fluctuate under different loadings, a static test was performed with a load of 25 g to limit the indentation diagonals. The selected duration for maintaining this load was 15 s, and the results were acquired from the instrument directly.

## 3. Analysis of the Melt Pool Shapes and Grain Refinement

The laser surface remelting experiments were performed on the top surfaces of the Al-Si alloy samples with a focus on understanding the modifications induced by the laser treatment. The areas of interest in this study were the sections that underwent remelting due to the laser’s path.

**Figure 2:**
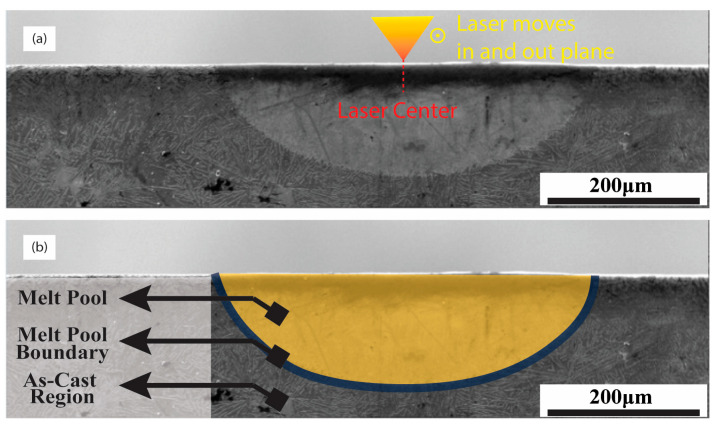
SEM image of the melt pool indicating the difference in the fibrous Si density and distribution in the remelted area (light gray) compared to the as-cast material. (**a**) The position of the laser center and the direction of the laser move in and out, while (**b**) melt pool, melt pool boundary, and the as-cast region are delimited with no microtrichial changes.

To examine these remelted sections more closely, cross-sectional cuts were prepared, revealing a semi-elliptical shape of the modified regions, as presented in Figure 2.

**Figure 3:**
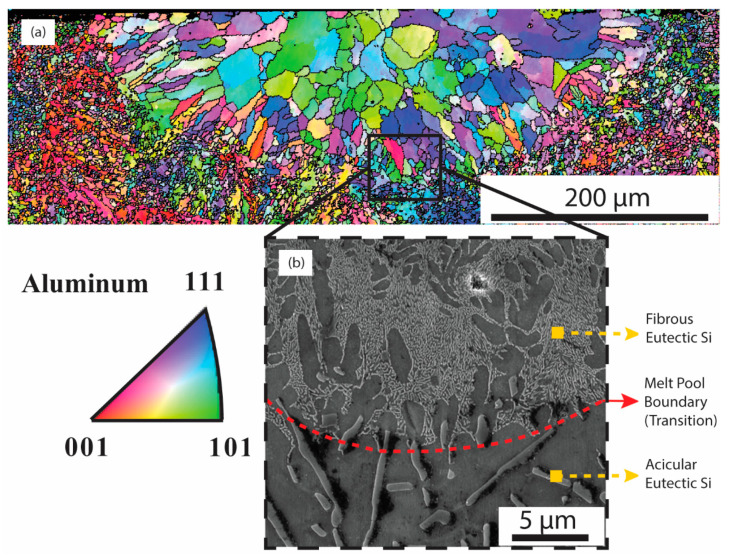
Grain size and their orientation in the melt pool (Experiment #5, Table 1) showing the transition zone from the molten area to the as-cast section of Al-20Si. (**a**) The EBSD of the melt pool and the transition zone to the as-cast section and (**b**) the morphology of the dendritic fibrous Si in a selected area and indicated in (**a**) through a black solid contour line.

The molten zones were analyzed using SEMs and performed EBSDs. Figure 3a shows the results of the Electron Backscatter Diffraction (EBSD) analysis of the aluminum grains within the remelted area. Notably, the grains are larger in the center of the melt pool, indicating a coarser granular structure, while they appear to be more finely structured close to the edges, suggesting a rapid solidification effect. 

According to Abboud and Mazumder [17], the microstructure and properties of hyper-eutectic Al-Si alloys after undergoing laser remelting impact the size of the aluminum grains (Al size), spacing or distance between the adjacent aluminum grains (Al spacing), and the size of the silicon particles within the microstructure of the hyper-eutectic Al-Si alloy (Si size). 

These parameters are important because they influence various mechanical properties such as strength, ductility, and toughness. Laser remelting processes can influence the spacing between aluminum grains, affecting the overall microstructural refinement and performance of the alloy. Thus, laser surface remelting caused a series of microstructural reductions in melt pools: Al colony size change (Figure 3b), Al spacing changes, and Si size and morphology changes. The as-cast microstructure which is seen below the molten area exhibits acicular or needle-like silicon in distinct brighter hues as observed in Figure 3b. This figure also emphasizes the transition zone between the melted region and the untouched section (the as-cast alloy), with a dashed line highlighting this boundary. Outside of the melted area, silicon structures, approximately 10 μm in size, are discernible. Contrarily, inside the melt pool, the silicon structures are substantially smaller, adopting a fibrous eutectic form and measuring no more than 150 nm in diameter. Eutectic Al-Si zone and Al dendrites (possibly containing Si precipitates [18]) were observed as well.

**Figure 4:**
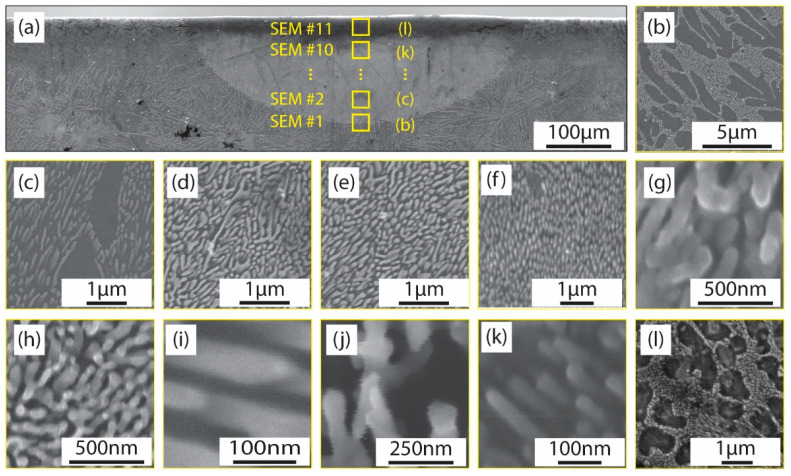
Microstructure refinement in the melt pool; (**a**) the representation of the melt pool (Experiment #5, Table 1) and the location of the 11 samples collected for microscopy analysis; (**b**–**l**) fibril silicon evolution at different depths from the contact between the laser and the material.

To closely examine the dynamics of the melting and solidification processes, the melt pool was analyzed by segmenting it into 11 distinct zones based on depth, enabling a more granular study of its characteristics. This systematic division and the detailed observation of each zone are illustrated in Figure 4. This division is designed to facilitate a zone-by-zone investigation of the changes within the pool, particularly focusing on the refinement of the silicon fibers.

**Figure 5:**
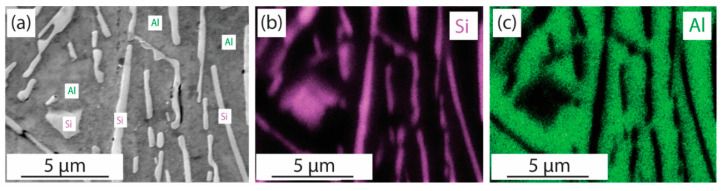
EDX analysis of the as-cast material showing the presence of large fibrous Si playing the role of a spacing for the Al matrix; (**a**) Si fibers in Al matrix, (**b**) Si atoms in purple, (**c**) Al atoms in green.

An additional analysis of the Al spacings and Si fiber diameters in the acquired SEM images (labeled b through l) was conducted using an external image processing script. The verification of the existence of Si fibrous was carried out through an EDX analysis on the as-cast material shown in Figure 5. 

To determine the change in the density of these fibrous Si, the spacing between the Si fibers in 11 locations marked in Figure 4 was quantified and it is summarized in Table 2. Moreover, the diameter of the Si fibers considering their orientation with respect to the measuring direction was measured in the same locations and it is summarized in Table 3.

## 4. Dimensional Analysis and Predictions

### 4.1. Correlation of the Microstructure Refinement and Cooling Rate

**Figure 6:**
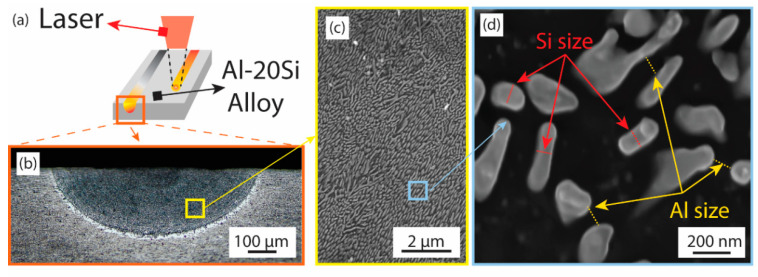
(**a**) General view of a laser surface remelting experiment; (**b**) the cross-section of a melt pool; (**c**) fully eutectic microstructures in a particular region of the melt pool; (**d**) a detailed view of the microstructures. The Si fiber diameter and Al spacing can be read from the red and yellow dashed lines, respectively.

**Figure 7:**
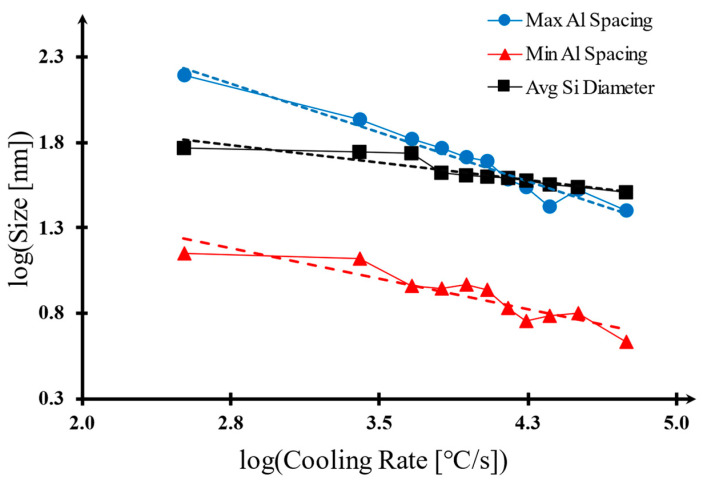
The relationship between the cooling rate and microstructure size on a logarithmic scale. The dashed lines indicate trendlines.

In this section, the microstructure of the laser-remelted Al-20Si (Figure 6a) was primarily characterized with regard to the Si size (the Si fiber diameter shown as red dashed lines in Figure 6d) and the distances between the nano Si phases, also referred to as the aluminum spacing (yellow dashed lines in Figure 6d). An exemplary measurement is depicted in the following Figure 6b–d:

In Figure 6d, the red arrows indicate the measurements of the nano Si formations in the brighter phases. The darker background signifies the Al spacing, otherwise known as the Al matrix, which occurs between the Si phases. This was also measured, as represented by the yellow arrow. Following the collection, basic statistical data—including the maximum, minimum, average, and standard deviation—were derived. Figure 7 presents a comparative analysis of two of the obtained datasets.

It is evident that both the Si fiber diameter and Al spacing between these fibers have been significantly diminished with the laser processing. This notable change can be credited to the thermal history within the melt pool. As the solidification time decreases (or alternatively, as the cooling rate increases), there is less time for particles to nucleate. When these microstructure dimensions are studied further, it is possible to establish a relationship between depth from the surface and microstructure size due to the cooling rate effect [7]. Such a relationship is depicted in Figure 7, where the cooling rate has been calculated using the Rosenthal equation provided in equation #2. To determine the correlations between R and these two categories of parameters, numerical studies are performed. 

**Figure 8:**
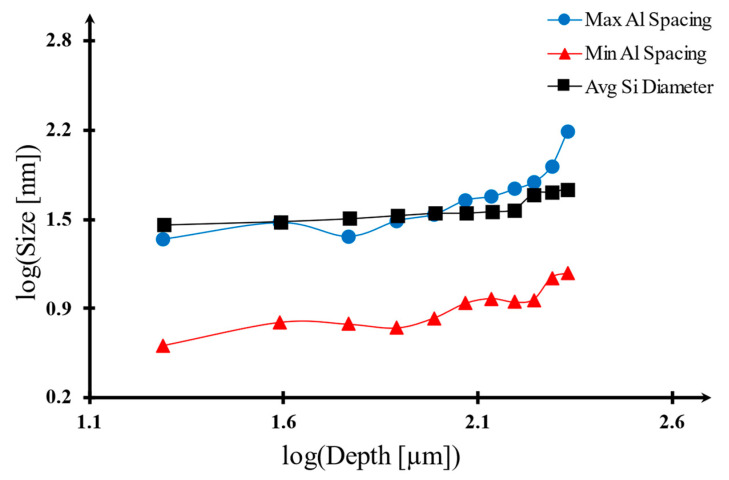
Microstructure size changes with changing depth. As depth increases, microstructures tend to be larger.

Although the overall statistical data does not indicate any strong correlation, there are two points where this correlation seems evident: the average Si fiber diameters ($\lambda_{Si}$) as related to depth, and the maximum and minimum Al spacing ($\lambda_{Al}$) as presented in Figure 8. The curve-fitting equations for these correlations along with their corresponding regression factor R^2^ values are provided as follows:

- $\lambda_{Al}^{max}$: 0.613x + 0.426 (R^2^ = 0.71)
- $\lambda_{Al}^{min}$: 0.439x − 0.018 (R^2^ = 0.82)
- $\lambda_{Si}^{avg}$: 0.231x + 1.116 (R^2^ = 0.74)

In this context, ‘x’ is equivalent to the base 10 logarithm of depth measured in micrometers (µm)

As investigated by Mazumder et al., it has been established that microstructural refinement, by itself, significantly enhances material strength. For instance, while the yield strength for as-cast Al-33.2Cu (wt.) alloy ranges between 200 and 400 MPa, this value increases to approximately 1100 MPa following laser processing [8]. Although additional research is required to determine the compressive and/or tensile behavior of the laser-remelted Al-20Si, we anticipate an increase in the mechanical response from our studies, especially when the extended solid solubility due to laser processing is considered [19]. However, it is important to note that the nanomechanical response is highly contingent upon the specific location from which the test sample is taken. This could be within a specific nano eutectic colony, polycrystalline nano eutectics, or a heterogeneous structure of Al dendrites and fibrous Si eutectics.

### 4.2. Correlation of the Melt Pool Dimensions and Energy Density

The dimensions of the melt pool, which provide critical insights into the laser processing conditions, were determined through the measurements obtained from Scanning Electron Microscopy (SEM) for each experiment. The experiments conducted and their corresponding melt pool sizes are compiled in Table 4.

Understanding the dimensions of the melt pool is significant, as they offer a glimpse into the laser–material interaction during processing. By analyzing the melt pool size, we can deduce information about the heat input and thermal history experienced by the material. Such data are invaluable, particularly when confirming the accuracy of thermal models, whether they are based on analytical or numerical approaches.

Additionally, the volume of the melt pool indicates the quantity of material affected by the laser process. Knowing the extent of the processed material is essential when considering the scalability of laser processing techniques. For industrial applications, the accurate estimation of the final dimensions of the melt pool is integral to ensuring consistency and efficiency in production. This knowledge may also aid in predicting and controlling the mechanical and physical properties of the treated material, making it an important parameter in laser processing research and applications.

Energy density for this table was calculated by means of Equation (1):

(1) $$E\;\left[J/{\mathrm{mm}}^{3}\right]=\frac{Q\;\left[W\right]}{A\;v\;\left[{\mathrm{mm}}^{3}/s\right]}$$

where Q is the laser power, A is the area of the laser spot on the surface, and v is the laser scanning velocity. 

A robust correlation has been observed between energy density and the dimensions (width and depth) of the melt pool, particularly when power inputs are comparable. This relationship is underscored by the exceptionally high R^2^ values corresponding to the provided power regression equations, both exceeding 99%.

The regression analysis for the depth and width dimensions as a function of energy density results in high R^2^ values. These data can be utilized to estimate the melt pool radius for a specified material, and potentially applied to other metal alloys. Rosenthal [20] proposed the following equation for the simple estimation of melt pool dimension, Equation (2):

(2) $$\;T-T_{0}=\frac{\varepsilon q}{2\pi K}\exp\left[\frac{-v}{2\;\alpha }\;\xi \right]\frac{1}{R}\exp\left[\frac{-v}{2a}R\right]$$

where R is the radius of the melt, T is laser temperature (°C), T_0_ is the initial temperature (°C), ξ is the location of the T temperature (m), α is thermal diffusivity (m^2^/s), ɛ is the laser absorb efficiency, K is thermal conductivity (W/mK), v is the laser speed (m/s), q is the laser power (W), a is the laser spot area (mm^2^).

**Figure 9:**
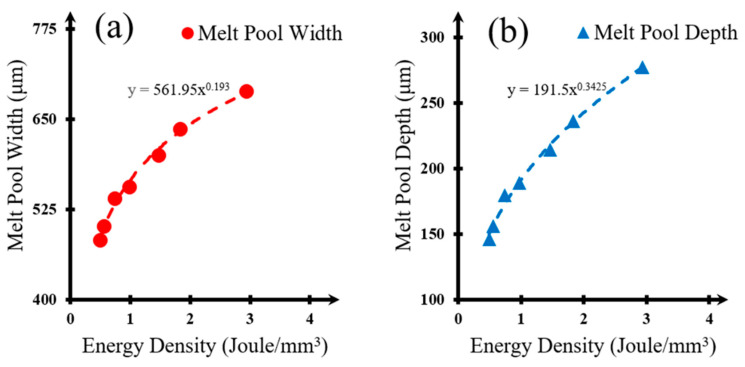
Melt pool dimensions with respect to energy densities. Corresponding regression factors, R^2^, are 0.992 and 0.991; (**a**) melt pool width variation with energy density, and (**b**) melt pool depth variation with energy density.

Rosenthal [19] empirically determined that R is dependent on the width and the depth of the molten pool through an empirical relationship:

R = ((w/2)^2^ + h^2^)^0.5^ (3)

where w is the width (mm) and h is the depth (mm). R is also dependent on physical material properties such as absorptivity, thermal conductivity, and thermal diffusivity on one hand and process parameters such as laser power, scanning speed, and the temperature domain on the other hand. In this study, the R values from Figure 9 were used in Equations (4) and (5) as follows:

(4) $$\;R^{\prime }=\frac{\varepsilon Q}{2\pi K}\frac{1}{T-T_{0}}\exp\left[C\;\xi \right]\exp\left[C\;R\right]$$

(5) $$\;R=\sqrt{{\left(280.975{\;x}^{0.193}\;\right)}^{2}+{\left(191.5{\;x}^{0.3425}\;\right)}^{2}}\;10^{-6}$$

$\mathrm{where}\;x\;\mathrm{is}\;\mathrm{the}\;\mathrm{energy}\;\mathrm{density}\;\left(\frac{J}{{\mathrm{mm}}^{3}}\right),\;C=-\frac{v}{2a}$, ε is laser absorption efficiency, ξ is the location of the temperature value, v is laser scanning speed, α is thermal diffusivity, T is current temperature, T_0_ is room temperature, Q is laser power, and K is thermal conductivity. As Equation (4) is indefinite where energy density is equal to zero, or T equals to T_0_, the following graph has been presented to circumvent such extremes.

In Equation (4), T represents the input temperature, which is critical for obtaining melt pool dimensions. To accurately determine these dimensions, one can consider the maximum and minimum temperature values based on the point at which melting initiates. The chemical composition of our material varies locally, with a silicon content ranging from 0 to 20 weight percent. According to the equilibrium phase diagram, the melting point of pure aluminum is 594 °C, while the melting point for an aluminum alloy with 20% silicon is 820 °C. Consequently, the results derived from using these two distinct temperature values are referred to as the upper limit and lower limit for the melt pool shapes in this study.

**Figure 10:**
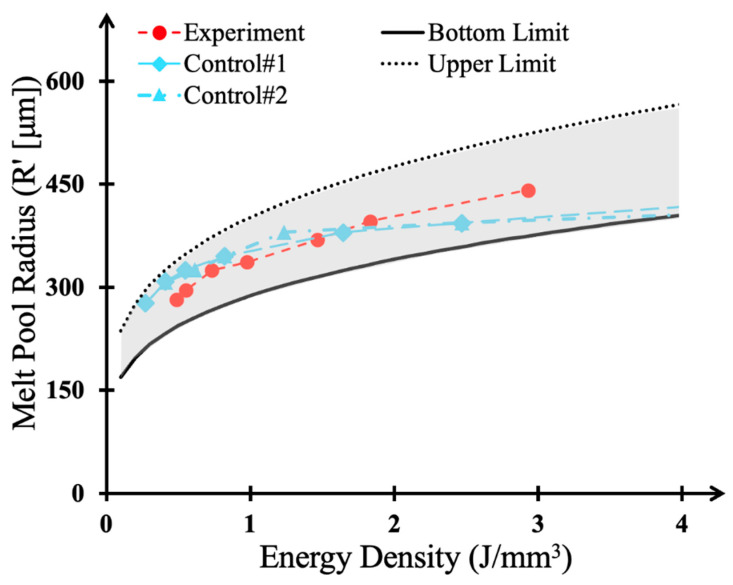
Melt pool radius variation with the laser remelting energy density.

As shown in Figure 10, the changes observed in the experiment, indicated by the red dots, fall within the expected estimation domain, represented by the gray shaded area. These results are consistent with an additional set of experiments conducted at the laser powers of 1200 W (illustrated by blue squares) and 1800 W (illustrated by blue triangles), further corroborating the robustness of the proposed model.

### 4.3. Prediction of the Melt Pool Dimensions Based on Plasma Signals

**Figure 11:**
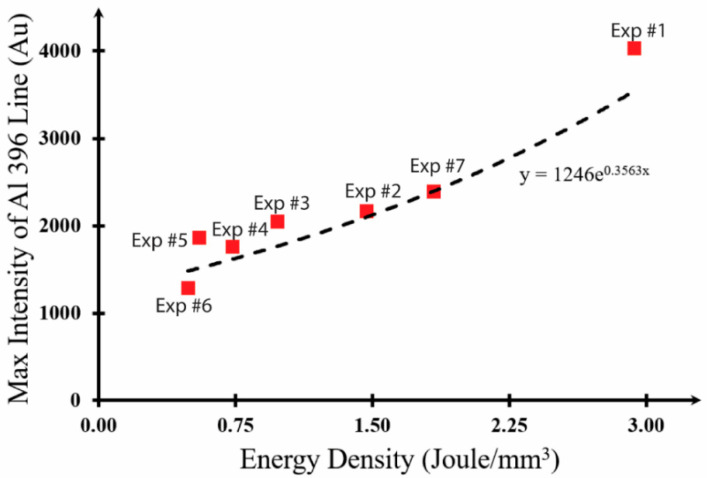
Spectral light intensity changes with respect to energy densities.

Energy input and plasma intensity are reported to have a direct relationship, with plasma intensity calculated based on the population of atoms in the upper state [21]. With the results obtained from the experiments conducted in this study, this can be easily demonstrated as shown in Figure 11. As a result, a detailed examination of the dimensions of the melt pool (which correlate almost perfectly with energy density, as shown in Figure 10) and spectral data could potentially reveal a strong connection. Furthermore, it is important to highlight that the cross-sectional view used to characterize the melt pool in this study was taken right from the start of the laser–material interaction. Even though the total length of the scanned zone was 22.25 mm, the cross-sectional SEM photo was obtained approximately 0.5 mm and 1 mm from the beginning. Therefore, the plasma signals from the first five frames (specifically 0–50 ms) should be factored in. The following figure also presents these data alongside the regression fit.

The plasma intensity from the process can be used to estimate the melt pool depth. Knowing the melt pool depth, the melt pool width can also be found as they are shown in good agreement. This allows for the projection of the total area treated by the laser within a considerably short amount of time. By simply observing the spectral responses from the processes, an operator can adjust processing parameters (laser power, etc.) during the operation. The relationship between melt pool dimensions and the 396 nm wavelength spectral intensity (x) is approximated with the following equations:

w/h = −0.759ln(x) + 8.7269 (regression factor R^2^ = 0.850) (6)

h = 124.89ln(x) − 755.58 (regression factor R^2^ = 0.884) (7)

**Figure 12:**
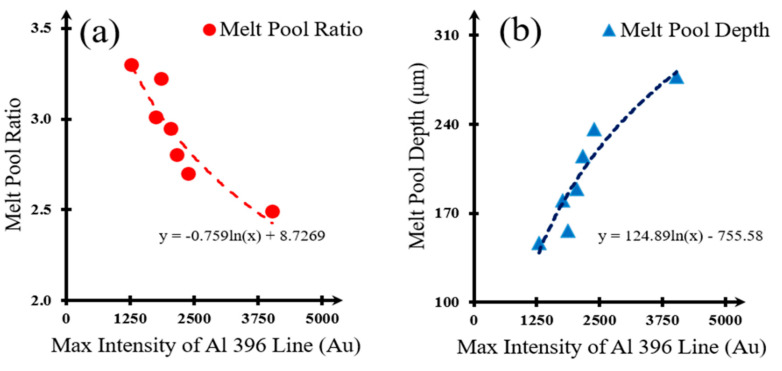
Melt pool dimension changes are closely related to the maximum intensity of Al 396 nm signal. Here, the melt pool ratio is defined as the ratio of the width to depth of the melt pool; (**a**) melt pool ratio at variation of the maximum intensity of Al 396 line, and (**b**) melt pool depth at variation of the maximum intensity of Al 396 line.

Melt pool to the melt pool depth (w/h) and depth h are dependent on the maximum intensity (Figure 12a,b). The regression form based on the experimental data are given by the following Equations (6) and (7).

## 5. Conclusions

In the present study, it has been shown that there is a strong correlation between Al and Si microstructure size and their relative depth with respect to the surface of the melt pool.

As the distance from the surface increases, the dimensions of the microstructure also increase. That implies the cooling rate on the surface is highest since it is the driving factor for microstructure formation.

In the eutectic region of experiment #2, we determined the composition to be 17.55% silicon by weight, hypothesized to be due to the refinement of aluminum spacing and the super saturation of eutectic structures.

Significant refinement in the Al-Si alloy microstructures and atom trapping because of a very high cooling rate led to a considerable increase in hardness, up to twice the initial measure.

The melt pool dimensions and energy density showed an almost perfect relationship as the trend in their regression equations had an R^2^ value greater than 99%. These calculated dimensions could prove valuable in predicting new melt pool dimensions for any material based on their respective thermo-physical properties and process conditions.

Due to the nature of emission spectroscopy, an increase in the input energy (in this context, energy density) results in a considerable increase in the intensity of spectral emission. Therefore, the correlation between the melt pool dimensions and spectral intensity could be effectively utilized for real-time dimension prediction.

## Figures and Tables

**Table 1:** Laser remelting parameters used in experiments.

Experiment Number	Laser Power (W)	Laser Scanning Speed (mm/s)
#1	1200	30
#2	1200	60
#3	1200	90
#4	1200	120
#5	1200	160
#6	1200	180
#7	1500	60
Control #1	1200	10–300
Control #2	1800	10–300

**Table 2:** Aluminum spacing values within the melt pool.

Depth (μm)	SEM Image Number	Min. (nm)	Max. (nm)	Mean (nm)
213.95	1	12.73	139.39	36.29
194.50	2	11.80	77.32	23.78
175.05	3	8.21	59.09	22.13
155.60	4	7.87	52.34	15.70
136.15	5	8.34	46.36	15.30
116.70	6	7.70	43.50	16.76
97.25	7	6.05	34.30	15.34
77.80	8	5.12	30.73	14.36
58.35	9	5.45	23.61	12.65
38.90	10	5.62	29.83	22.55
19.45	11	3.82	22.47	10.94

**Table 3:** Silicon fiber diameter values within the melt pool.

Depth (μm)	SEM Image Number	Min. (nm)	Max. (nm)	Mean (nm)
213.95	1	18.55	105.09	52.39
194.50	2	17.97	82.66	49.60
175.05	3	23.70	96.38	48.37
155.60	4	20.03	71.02	37.32
136.15	5	17.92	80.66	36.05
116.70	6	18.26	62.08	35.19
97.25	7	14.18	109.35	35.03
77.80	8	17.05	56.26	33.75
58.35	9	15.93	47.80	32.09
38.90	10	10.02	68.48	30.65
19.45	11	15.95	77.08	29.00

**Table 4:** Experiment numbers and corresponding melt pool dimensions in terms of width, depth, and ratio.

Exp. No.	Energy Density (J/mm^3^)	Melt Pool Width (μm)	Melt Pool Depth (μm)	Melt Pool Ratio (Width/Depth)
1	2.934	689.07	277.15	2.49
2	1.467	600.27	214.52	2.80
3	0.978	556.59	189.19	2.94
4	0.734	540.78	179.77	3.01
5	0.550	501.90	156.03	3.22
6	0.489	482.44	146.33	3.30
7	1.834	636.75	236.34	2.69

## Data Availability

No new data were created or analyzed in this study. Data sharing is not applicable to this article.

## Nomenclature

**Symbol**	**Meaning**
T	Temperature (°C)
T_0_	Initial Temperature (°C)
ξ	Location of the T Temperature (x − vt) (m)
α	Thermal Diffusivity (m^2^/s)
ɛ	Laser Absorb Efficiency
K	Thermal Conductivity (W/mK)
R	Melt Pool Radius [ξ^2^ + x^2^ + y^2^]^1/2^
v	Laser Speed (m/s)
Q	Laser Power (W)
A	Laser Spot Area (mm^2^)

## References

[1] Sahu H., Dave R., Chauhan S., Dwivedi R. (2024). Review on Laser Welding of High Strength Aluminium Alloy for Automotive Applications. SAE Tech. Pap. Ser..

[2] Li S.S., Yue X., Li Q.Y., Peng H.L., Dong B.X., Liu T.S., Yang H.Y., Fan J., Shu S.L., Qiu F. (2023). Development and Applications of Aluminum Alloys for Aerospace Industry. J. Mater. Res. Technol..

[3] Stemper L., Tunes M.A., Tosone R., Uggowitzer P.J., Pogatscher S. (2022). On the Potential of Aluminum Crossover Alloys. Prog. Mater. Sci..

[4] Kamarska K., Dimova D., Dochev B., Dubaradjieva M., Peeva N., Demirev T. (2024). Investigation of the Corrosion Resistance of Complex Alloyed Hypereutectic Aluminium-Silicon Alloys. AIP Conf. Proc..

[5] Dinesh Kumar P.K., Darius Gnanaraj S. (2024). Studies on Al-Si Based Hybrid Aluminium Metal Matrix Nanocomposites. Mater. Today Commun..

[6] Tenekedjiev N., Gruzleski J.E. (2016). Hypereutectic Aluminium-Silicon Casting Alloys—A Review. Cast Metals.

[7] Kayitmazbatir M., Lien H.H., Mazumder J., Wang J., Misra A. (2022). Effect of Cooling Rate on Nano-Eutectic Formation in Laser Surface Remelted and Rare Earth Modified Hypereutectic Al-20Si Alloys. Crystals.

[8] Lei Q., Ramakrishnan B.P., Wang S., Wang Y., Mazumder J., Misra A. (2017). Structural Refinement and Nanomechanical Response of Laser Remelted Al-Al2Cu Lamellar Eutectic. Mater. Sci. Eng. A.

[9] Wei B., Wu W., Ghosh A., Kayitmazbatir M., Misra A., Wang J. (2023). In Situ SEM Characterization of Tensile Behavior of Nano-Fibrous Al–Si and Al–Si–Sr Eutectics. J. Mater. Sci..

[10] Song L., Mazumder J. In-Situ Spectroscopic Analysis of Laser Induced Plasma for Monitoring of Composition during Direct Metal Deposition Process. Proceedings of the 29th International Congress on Applications of Lasers and Electro-Optics, ICALEO 2010—Congress Proceedings 2010.

[11] Choi J., Wooldridge M., Mazumder J. (2023). Spectroscopy-Based Smart Optical Monitoring System in the Applications of Laser Additive Manufacturing. J. Laser Appl..

[12] Choi J., Mazumder J., Rice A. (2020). Innovative Additive Manufacturing Process for Successful Production of 7000 Series Aluminum Alloy Components Using Smart Optical Monitoring System. SAE Technical Papers.

[13] Song L., Wang C., Mazumder J. (2012). Identification of Phase Transformation Using Optical Emission Spectroscopy for Direct Metal Deposition Process. High Power Laser Materials Processing: Lasers, Beam Delivery, Diagnostics, and Applications.

[14] Sun W., Zhang Z., Ren W., Mazumder J., Jin J.J. (2022). In Situ Monitoring of Optical Emission Spectra for Microscopic Pores in Metal Additive Manufacturing. J. Manuf. Sci. Eng. Trans. ASME.

[15] Lu Y., Sun G., Xiao X., Ren W., Sprague E., Ni Z., Mazumder J. (2021). In Suit Monitoring of Solidification Mode, Porosity and Clad Height during Laser Metal Deposition of AISI 316 Stainless Steel. J. Manuf. Process.

[16] Ren W., Mazumder J. (2020). In-Situ Porosity Recognition for Laser Additive Manufacturing of 7075-Al Alloy Using Plasma Emission Spectroscopy. Sci. Rep..

[17] Abboud J., Mazumder J. (2020). Developing of nano sized fibrous eutectic silicon in hypereutectic Al–Si alloy by laser remelting. Sci. Rep..

[18] Lien H.H., Mazumder J., Wang J., Misra A. (2020). Microstructure Evolution and High Density of Nanotwinned Ultrafine Si in Hypereutectic Al-Si Alloy by Laser Surface Remelting. Mater. Charact..

[19] Wei B., Wu W., Xie D., Lien H.H., Kayitmazbatir M., Misra A., Wang J. (2021). In Situ Characterization of Tensile Behavior of Laser Rapid Solidified Al–Si Heterogeneous Microstructures. Mater. Res. Lett..

[20] Rosenthal D. (1946). The Theory of Moving Sources of Heat and Its Application to Metal Treatments. J. Fluids Eng..

[21] Shin J., Mazumder J. (2016). Plasma Diagnostics Using Optical Emission Spectroscopy in Laser Drilling Process. J. Laser Appl..

